# Effects of a Trans-Galactooligosaccharide on Biochemical Blood Parameters and Intestine Morphometric Parameters of Common Carp (*Cyprinus carpio* L.)

**DOI:** 10.3390/ani10040723

**Published:** 2020-04-21

**Authors:** Ewa Ziółkowska, Joanna Bogucka, Agata Dankowiakowska, Mateusz Rawski, Jan Mazurkiewicz, Magdalena Stanek

**Affiliations:** 1 Department of Animal Physiology, Physiotherapy and Nutrition, Faculty of Animal Breeding and Biology, UTP University of Science and Technology, Mazowiecka 28, 85-004 Bydgoszcz, Poland; bogucka@utp.edu.pl (J.B.); agata.dankowiakowska@utp.edu.pl (A.D.); winiarska@utp.edu.pl (M.S.); 2 Division of Inland Fisheries and Aquaculture, Faculty of Veterinary Medicine and Animal Science, Poznań University of Life Sciences, Wojska Polskiego 71C, 60-625 Poznań, Poland; mateusz.rawski@up.poznan.pl (M.R.); jan.mazurkiewicz@up.poznan.pl (J.M.)

**Keywords:** blood, fish, histology, intestine, parameters, prebiotic

## Abstract

**Simple Summary:**

Prebiotics are important feed additives used in aquaculture. These are substances that are breeding grounds for beneficial bacteria and that inhibit the development of pathogens; accelerate healing and regeneration of the intestinal epithelium; increase mucus production; help maintain normal pH in the intestine; limit the growth of pathogenic bacteria; increase calcium, iron and magnesium absorption; and also have beneficial effects on glucose and protein metabolism in the liver. The saccharide-based prebiotic used in the carp experiment had a positive effect on intestine morphometric parameters. Supplementation of this prebiotic had no negative effect on growth performance and did not disturb the homeostasis of the fish, as demonstrated by the values of biochemical blood parameters.

**Abstract:**

The aim of the study was to evaluate the effects of a trans-galactooligosaccharide prebiotic (GOS) on the growth performance, biochemical blood parameters, and intestine morphometric parameters of common carp. The 60-day-long experiment was performed on one-year-old fish with a mean body weight of 180 g (±5 g). Three diets were used: control diet 1 (C) with no microbiota affecting feed additives, diet 2 (B1) with 1% of prebiotic, and diet 3 (B2) with 2% of prebiotic, in four replications (tanks) per treatment and 25 fish per tank. At the end of the trial, 16 individuals from each group were used for analyses. The study showed that GOS supplementation did not affect growth performance. In turn, the prebiotic had a positive effect on the development of the intestine, and increased the height, width, and surface of the villi in B1 and B2 groups. The content of phosphorus (P) was significantly higher in B1 group compared with B2 group, which indicated that 1% addition of prebiotic causes better absorption of P from the intestine. The other biochemical indicators—namely lipid, protein and hepatic parameters, insulin, and Ca—were not affected by GOS treatment, which may indicate similar metabolic balance of fish in each experimental group. Serum triiodothyronine (TT_3_) and glucose (stress markers) concentrations were not significantly different among treatments groups. GOS may be recommended as a feed additive for common carp due to its positive effects on fish physiology and development of the gastrointestinal tract. However, our results suggest that 1% diet supplementation causes satisfactory reactions for the abovementioned aspects in comparison to control or 2% supplementation.

## 1. Introduction

In all livestock, the composition of gut microbiota is one of the most important factors determining the proper growth and functioning of the animal body and preventing diseases. It is believed that the gut microbiome is crucial for both the digestion and assimilation of nutrients, as well as regulation of the immune response [1]. In aquaculture, where population density and environmental pressure increase the risk of alterations in gut health (disturbed intestinal homeostasis), many feed supplements are used for prophylaxis and normalization of the gut microbiome growth [2].

Prebiotics are a carbon and energy source for both physiological gut microorganisms and beneficial ones contained in probiotics supplied with feed. Prebiotics contain a range of substances that promote the growth and proliferation of lactic acid bacteria and play a very important role in inhibiting the growth of pathogenic gut microbial flora while stimulating microorganisms beneficial for the animal host (e.g., fructooligosaccharides (FOS), transgalactooligosaccharides (t-GOS), inulin, and mannanooligosaccharides (MOS) [3]. An immunostimulating effect of prebiotics has also been reported, including their ability to activate T and B lymphocytes, which increase the immune resistance of the body to pathogenic microorganisms [4,5,6]. Hoseinifar et al. [7] confirmed that prebiotics can significantly increase the leukocyte count and improve the resistance of animals to stress. 

One of the most comprehensive methods of assessing the health and welfare of the animal, as well as the proper function of individual internal organs, relies on the analyses of hematological and biochemical parameters of blood. These parameters can be used to assess the homeostasis of the body, the nutritional status, and to detect possible symptoms of a disease [8]. The most important analyzed parameters are: total protein (TP), albumin, globulin and alanine aminotransferase (ALT), aspartate aminotransferase (AST), alkaline phosphatase (ALP), total cholesterol (TC), low-density lipoprotein (LDL), high-density lipoprotein (HDL), urea, triglycerides (TG), bilirubin and creatinine [9,10,11]. glucose, cortisol, and thyroid hormones T_3_ and T_4_, which are important indicators of stress [12].

However, the histological analysis of the gastrointestinal tract is necessary for the complete assessment of digestion and absorption of food. Thus, histological examination of the digestive system, especially of the intestine, is crucial regarding prebiotic use [13]. Important parameters are the height and width of the villi and depth of the crypt, as demonstrated by Asaduzzaman et al. [14] for Malaysian mahseer (*Tor tambroides*), Akter et al. [15] for striped catfish (*Pangasianodon hypophthalmus*), Qiyou et al. [16] for hybrid sturgeon (*Acipenser schrenckii* × *Huso dauricus*), Yang et al. [17] for tilapia (*Oreochromis niloticus* × *O. aureus*), Korylyak [18] for common carp, and Zhang et al. [19] for Koi carp (*Cyprinus carpiod*). Larger villi mean more absorptive surfaces and higher digestive enzymes, which are supposed to increase nutrient absorption and fish growth. A review of the literature shows positive effects of trans-galactooligosaccharide supplied in different doses on growth performance parameters, blood biochemical indices, and intestinal microstructure in carp aquaculture. The results demonstrate that prebiotics usually improve growth factors (final body weight, food conversion ratio, and protein efficiency ratio) [20]. In one study, the Trans-Galactooligosaccharide (GOS) -treated group (obtained from lactose) had significantly higher total protein and serum lysozyme activity than other treatments (*p* < 0.05) [21]. Analyses carried out by Mehrabi et al. [22] conformed that dietary inclusions of GOS (Primalac^®^, and to a lesser extent Immunowall^®^, or their mixture) positively affect most of the parameters examined in the experimental *C. carpio*, leading to improved growth performance, enhanced body composition, and stimulated fish immune system.

Prebiotics used in the experiments (trade name Bi^2^tos, Clasado Biosciences Ltd., Jersey, UK) were manufactured via enzymatic transgalactosylation of milk lactose by the whole cells of *Bifidobacterium bifidum* 41171. For this reason, Bi^2^tos specifically promotes growth of *Bifidobacterium* spp. [23]. The genome of *Bifidobacterium* spp. encodes carbohydrate-degrading enzymes with high affinity to GOS [24]. In fermentation experiments carried out by Tzortzis et al. [23], *B. bifidum* showed an increased preference towards the produced galactooligosaccharide mixture, displaying higher growth rate and short-chain fatty acid production when compared with commercially available oligosaccharides. Bi^2^tos delivered in ovo on day 12 of egg incubation increased the lactobacilli and bifidobacteria fecal counts of 1-day-old chicks [25]. Sławińska et al. [26] showed the effects of microbiota modulation with in ovo stimulation in adult broiler chickens. Bi^2^tos was used in projects investigating the microstructure of a broiler chicken small intestine and the results confirmed a significant effect of this prebiotic on the histomorphology of this tissue and an effect on gut structure, which should contribute to improvement in nutrient absorption in the gut [27,28].

The aim of this study was to analyze the effects of dietary supplementation with 1% and 2% trans-galactooligosaccharide (GOS) on growth performance, selected biochemical blood parameters, and intestine morphometric parameters, and to verify which GOS dose may positively affect good health, proper development of the intestine, and growth performance parameters of common carp.

## 2. Material and Methods

### 2.1. Fish Culture and Feeding

The study was carried out in strict accordance with the recommendations of the National Ethics Commission (Warsaw, Poland). All procedures and experiments complied with the guidelines of the Local Ethics Commission of the Poznań University of Life Sciences (Poznań, Poland) with respect to animal experimentation and care of animals under study, and all efforts were made to minimize suffering according to Polish law and the EU Directive (no. 2010/63/EU) [29]. All members of the research team were trained in animal care, handling, and euthanasia by the Polish Laboratory Animal Science Association (PolLASA).

The diets were formulated according to common carp nutritional requirements. All experimental diets were processed by extrusion using a single-screw warm extruder (Metalchem S-60 Gliwice, Poland). The extrusion conditions were as follows: a 90 °C cylinder temperature in the zone of increasing pressure, a 100 °C cylinder temperature in the zone of high pressure, a 110 °C head temperature, a 52-rpm speed screw, and a 6-mm nozzle diameter. The diets were calculated as isonitrogenous (35% crude protein) and isoenergetic (18.5 MJ kg^−1^), with less than 4% crude fibers, and were formulated to meet carp nutritional requirements [30,31,32]. Three experimental diets were used: control diet 1 (C) without feed additives, diet 2 (B1), and diet 3 (B2) with 1% and 2% Bi^2^tos, respectively (Table 1).

**Figure 1:**
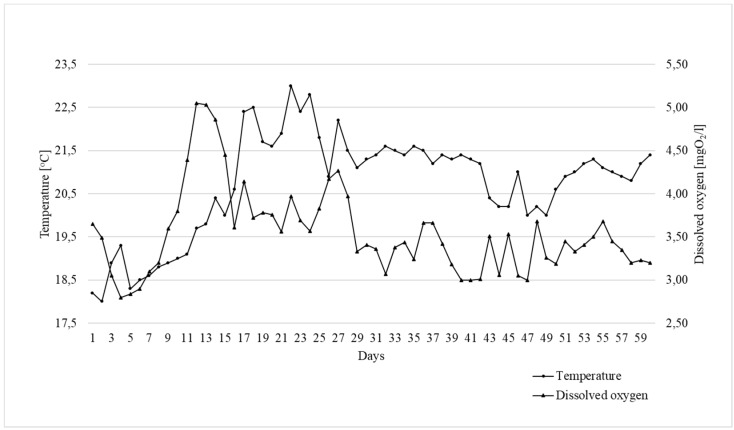
Changes in temperature (°C) and dissolved oxygen (mgO_2_/L) during trial.

The growth trial was done in the Experimental Station for Feed Production Technology and Aquaculture in Muchocin, Poland. Three-hundred one-year-old common carp (mean body weight 180 g) were used. The fish were randomly allocated into 12 concrete ponds (40 m^3^), with 25 fish per pond (according to Horváth [33]). The experiments were carried out in four replications. Each pond was equipped with an automatic band feeder allowing for the continuous supply of feed over 12 h per day. The calculated daily feed dose for each pond was given every day at 9:00 a.m., consumption was controlled visually twice a day, and the rate was corrected if needed. The daily feed dose was restricted to avoid feed loss, while the feeding rate was calculated with consideration of water temperature, current fish biomass, and consumption from the previous day, according to Miyatake’s [34] recommendations. A constant flow of water in the experimental system was ensured by an open flow system with a mechanical pre-filtration chamber. Control of water physio-chemical parameters (water temperature and content of oxygen solved in water) was carried out with the use of microcomputer oxymeter Elmetron CO-315 (Figure 1). The trial lasted 60 days. 

### 2.2. Growth Analyses

During the growth trial, all fish were weighed at 10-day intervals for feed dose control. The following calculations were done during the experiment:

Body Weight Gain (BWG) = W_2_ (g) − W_1_ (g) (1)

Feed Intake (FI) = feed intake (g)(2)

Feed Conversion Ratio (FCR) = FI (g)/BWG (g)(3)

Specific Growth Rate (SGR%) = 100(ln W_2_ − ln W_1_)/T (4)

Protein Efficiency Ratio (PER) = BWG (g)/protein intake (g)(5)

Percentage Weight Gain = (PWG/W_1_)·100% (6)

where W_1_ is the initial weight (g), W_2_ is the final weight (g), and T is the number of days in the feeding period.

At the end of the growth trial, each fish was individually weighed 24 h after the last feeding time. Then, four fish per pond were killed by decapitation after anesthesia (by immersion in 500 mg/L of MS-222 solution) and blood samples were collected post-mortem for further analyses. The number of fish was based on earlier studies performed by the authors [35,36] to give a necessary sample size for laboratory and statistical analysis and to avoid unnecessary animal sacrifice (according to 4R policy). All remaining animals were further kept in the experimental station.

### 2.3. Biochemical Analyses

Blood samples (*n* = 48) taken from the caudal vein were centrifuged after thrombus formation into obtain serum. Biochemical blood parameters were measured using a MINDRAY BS-120 biochemical analyzer and reference reagents from Stamar^®^ (Dąbrowa Górnicza, Poland). The analyzer was calibrated using a Multicalibrator 877UE in the presence of Qualinorm BS120 and Qualipath BS120. Insulin (µU/mL) and total triiodothyronine (TT_3_) (nmol/L) were analyzed with the DIAsource radioimmunoassay method using Ria-CT and INS-IRMA RIA kits (DIAsource Immunoassays S.A, Belgium) and a NZ-322 automatic sample changer with a gamma counter for ^125^I isotopes (Gamma Müvek, Hungary).

### 2.4. Histological Analyses

Samples of the proximal intestine for histological analyses (*n* = 48) (approximately 2 cm long) were taken from fish directly post-mortem. Individual segments of the intestine were rinsed with 0.9% normal saline and then preserved in 4% formalin buffered with CaCO_3_ solution. Preserved samples were dehydrated, cleared, infiltrated with paraffin in a tissue processor (Thermo Shandon, Runcorn, UK), and then embedded in paraffin blocks using a paraffin embedding system (Medite, Burgdorf, Germany). Paraffin blocks were cut on a rotary microtome (Thermo Shandon, Runcorn, UK) into 10 μm slices, which were then placed on microscope slides coated with chicken egg whites with the addition of glycerine. Specimens were deparaffinized, rehydrated, and then stained with the PAS (Periodic acid-Schiff) technique using the Schiff reagent for intestinal morphometric analysis. A Nikon Ci-L microscope integrated with a Nikon DS-Fi3 camera and NIS Elements software (Nikon Instruments Inc.) was used to measure the height and width of villi, depth of intestinal crypts, and thickness of the muscular layer. The height of the villi was measured for 10 randomly selected villi samples on the cross-section of a specimen. The length was measured from the top of the villus to its base at the ostium of the crypt. The width of the villus was measured at the mid-point of its length. The surface area of the villi was calculated based on the formula proposed by Sakamoto et al. [37]: (2π) × (VW/2) × (VH), where VW = villus width, and VH = villus height. The depth of intestinal crypt was defined as the depth of invagination between adjacent villi for 10 measured villi [38].

### 2.5. Statistical Analyses

Statistical calculations were made using STATISTICA 13.1 software (Dell, Round Rock, TX, USA, 2018). The arithmetic mean (x) and standard deviation (SD) were calculated. The normal distribution of data was confirmed using the Shapiro–Wilk test, while the homogeneity of variance was verified using the Levene test. Significant differences between the groups were tested with one-way analysis of variance (ANOVA), while Tukey’s test was used for multiple comparisons. The level of significance was determined at *p* ≤ 0.05.

## 3. Results

### 3.1. Growth Performance

The growth performance of common carp receiving feed supplemented with the prebiotic and control fish is presented in Table 2. Analyses showed no significant differences in the calculated parameters between the groups and no mortality was observed during the entire experimental period.

### 3.2. Biochemical Blood Parameters

The biochemical blood parameters measured in fish are presented in Table 3. Statistical analyses revealed a significant effect of prebiotic supplementation on the levels of phosphorus (P) (*p* < 0.05). The highest concentration of P was calculated in the samples of fish fed a diet with 1% prebiotic (group B1). The level of P was significantly higher in B1 group (13.62 mg/dL) than in B2 group (11.81 mg/dL). In terms of the protein profile (TP, albumin, globulin and urea), there were no significant (*p* > 0.05) differences among treatment groups. As analyses confirmed, lipid profile indicators (TC, TG, and NEFA) were not significantly (*p* > 0.05) affected by dietary GOS. Dietary supplementation of GOS had no stimulating effect on the thyroid hormone triiodothyronine (TT_3_) or glucose (stress marker) in the serum of carp. The content of TT_3_ was at a similar level in C, B1, and B2 groups. The same GOS effect was obtained for glucose and insulin. ALT and ALP activities did not significantly (*p* > 0.05) differ between treatments. Dietary inclusion of 1% or 2% GOS did not significantly affect (*p* > 0.05) Ca concentration.

### 3.3. Histological Measurements

Intestinal morphometric analyses demonstrated that the addition of GOS caused approximately 24% and 32% increases in villi height in groups B2 and B1, respectively (Table 4). Fish from the experimental groups also had significantly thicker villi (*p* < 0.05) compared to controls. Both the height and width of the villi correlated with their surface area, which was more than 50% greater in both groups of common carp receiving feed with the prebiotics. However, no significant differences in the depth of crypts were found between the analyzed groups. Our experiments also revealed that the supplementation of fish diet with the prebiotics significantly increased (*p* < 0.05) the Villi height (VH)/Crypt depth (CD) ratio, which may indicate the maturity of the intestinal mucosa.

**Figure 2:**
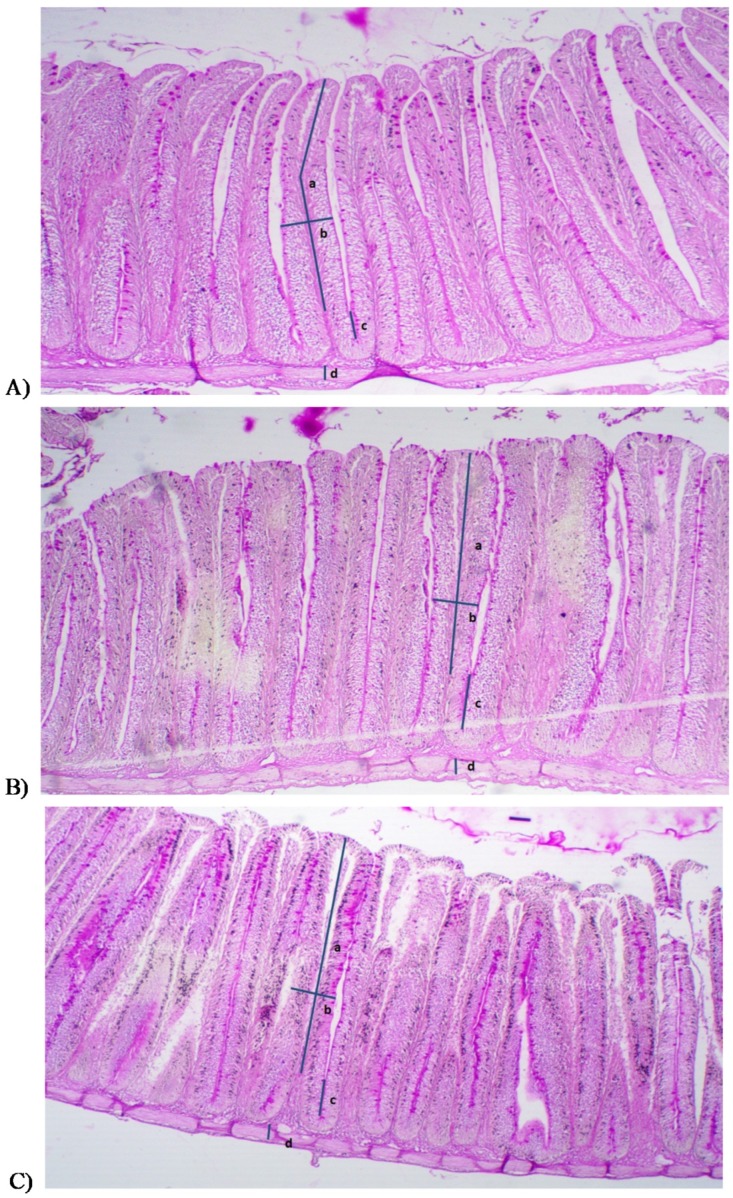
Light microscopy of the anterior portion of the intestine of common carp fed different prebiotic concentrations in the diet: (**A**) control group, (**B**) 1% Bi^2^tos^®^, (**C**) 2% Bi^2^tos^®^; a = villi height; b = villi width; c = crypt depth; d = tunica muscular thickness.

Moreover, fish receiving the diet with 2% GOS had a significantly thicker (*p* < 0.05) muscular layer compared to the control group. The results of histological measurements are shown in Figure 2.

## 4. Discussion

### 4.1. Growth

Similarly to our results, several studies confirmed no effect of prebiotic supplementation on the growth performance of various fish species [8,39,40]. This could be explained by the fact that the effect of prebiotics may vary depending on the solubility, fish species, and water temperature [20]. In addition, as Denji et al. [41] suggested, the lack of effects of prebiotics on the growth performance of common carp may be attributed to the inability of intestinal microbiota to ferment excessive levels of prebiotics. However, in the case of animals for which rearing or environmental conditions and feed composition are optimal, there may be no effect on the growth performance, indicating use of the full genetic potential. Thus, in such cases, the effects of feed additives—including prebiotics when animal welfare is maintained or no challenging dietary or pathogenic factors are used—should be further investigated. This was done in the present study to explain the mode of prebiotic action. In this case, the presented study blood biochemistry and histomorphological parameters were analyzed.

### 4.2. Blood Biochemistry

In this study, we confirmed that GOS supplementation affected phosphorus (P) levels in the serum of analyzed common carp. Significantly higher values of P in B1 group compared to B2 group indicate that dietary inclusion of 1% prebiotic causes better absorption of P from the intestine. Studies with pigs suggest that P may influence the immune system and the intestinal microbiota [42]. Research carried out by Pietrzak et al. [43] on the same group of fish confirmed that dietary supplementation of Bi^2^tos exerted immunomodulatory effects on skin mucosa, which was manifested by mRNA expression of the genes involved in cytokine, lysozyme, and acute-phase protein production, and which activated immunomodulatory pathways leading to gene expression modulation in skin-associated lymphoid tissues (SALT) of common carp.

There were no significant differences in the protein profile (TP, albumin, globulin) between GOS treatment and control groups, which may result from the same metabolic turnover manifested in a similar degree of utilization of feed nutrients and the same protein requirements for maintaining metabolic balance. As Ebrahimi et al. [44] reported, analyses of blood samples taken from common carp for the diet supplemented with Immunogen^®^ (Soroush Radian Co., Teheran, Iran), a prebiotic containing β-glucan and MOS (glucomannoproteins extracted from yeast *Saccharomyces cerevisiae*), revealed significant differences in the levels of TP (23.4–27.7 g/L), albumin (10.7–12.1 g/L), and globulin (12.7–15.6 g/L), and the increase in these parameters was positively correlated with doses of the prebiotic. Andrews et al. [45] observed significantly higher serum levels of TP and albumin in rohu fish (*Labeo rohita*) from the *Cyprinidae* family receiving feed with a MOS compared to the control. Better feed conversion of proteins from the food by the proteolytic action of prebiotics may be related to adherence of probiotics to intestinal mucosa and pathogen inactivation, along with modification of dietary proteins and bacterial enzymatic activity by intestinal micro flora, which influence gut mucosal permeability and regulation of the immune system [46,47].

As our analyses confirmed, GOS supplementation did not affect the lipid profile (TC, TG, and NEFA), which similarly to the protein profile, may indicate metabolic balance within all experimental groups. No significant differences in TC content between GOS-treated and control groups were found, as decreased cholesterol levels indicate a possible disease, an increased degree of physiological discomfort (stress), or a dysfunction of lipidic metabolism [48]. Our results are in line with Ye et al. [49], who reported that prebiotic (FOS and MOS) supplementation had no effect on the blood levels of TC and TG in Japanese flounder *(Paralichthys olivaceus*). Studies on mice using inulin as a prebiotic have shown that intestinal fermentation of fibers suppressed plasma and liver cholesterol, as well as triglyceride levels [50].

Similarly to our results for liver enzymes obtained for carp, analyses carried out by Ahmdifar et al. [51] and Hoseinifar et al. [11] for beluga (*Huso huso*) and Amani Denji et al. [41] for rainbow trout (*Oncorhynchus mykiss*) confirmed no significant differences in the levels of ALP or ALT between experimental groups. Because the increase in plasma AST and ALT may be connected to stress conditions, hepatocellular damages, or cellular degradation [3], we may conclude that the GOS has no adverse effect on the health status or condition of fish.

Prebiotics can increase the immune resistance (tolerance) of fish to stressors. Stress induces an increase in the levels of catecholamines (adrenaline and glucagon), which stimulate the glycogenolysis, leading to an increase in blood glucose levels. Glucose in serum is a major metabolite of carbohydrate metabolism. The amount of glucose in fish blood depends on the fish species or type, range from 25 to 350 mg/dL [3]. In our study, we confirm no significant differences in terms of glucose between GOS-treated and control groups. Similarly to our results, Silva et al. [52] reported constant values of glucose in the groups of tilapia supplemented with the probiotic *Bacillus amyloliquefaciens*, while Akrami et al. [3] reported similar results for MOS-treated (active mannan oligosaccharide^®^, Biorigin, Lencois Paulista, Säo Paulo, Brazil) beluga (*Huso huso*). These results may reflect the homeostatic state of fish against adverse conditions. Similarly to our results for the TT_3_ hormone (stress markers), no significant differences in levels of TT_3_ were observed by Adel et al. [53] for beluga sturgeon (*Huso huso*) fed with a prebiotic (brewer’s yeast), of by Mirghaed et al. [12] for rainbow trout (*Oncorhynchus mykiss*) fed with essential oil of *Eucalyptus* sp. compared to control groups.

### 4.3. Histology

Based on the conducted research, we confirm that dietary supplementation of GOS has contributed to the improvement of intestinal morphometric parameters by increasing the height and width of intestinal villi, which are responsible for the intestinal absorbent surface area. Both the height and width of the villi, which are correlated with their surface area, were significantly higher in GOS-treated groups compared to control. Similar results were obtained by Yuji-Sado et al. [54], who reported a positive effect of MOS supplementation on the intestinal microstructure in Nile tilapia (*Oreochromis niloticus*). In their study, fish fed 0.4% dietary MOS presented the highest (*p* < 0.05) intestinal fold height (430.27 ± 89.72 µm) compared to control, while fish fed 0.4% and 0.6% dietary MOS showed significant increases in muscular layer thickness (72.5 ± 21.95 µm and 71.44 ± 24.48 µm, respectively). It is worth noting that in the research by Zhou et al. [55], the height of microvilli in the proximal intestine was similar in groups supplemented with various prebiotic substances, including MOS, FOS, GOS, and galacto-gluco-mannans from hemicellulose extract (Previda™), and was significantly higher compared to the control group. As Anguiano et al. [56] revealed, GOS and MOS (Bio-Mos^®^, Alltech, Inc., Nicholasville, KY, USA) also significantly increased the height of microvilli in the anterior intestine in red drum (*Sciaenops ocellatus*) and hybrid striped bass (*Morone chrysops* × *M. saxatilis*) compared to the control group. However, the increase in prebiotic levels in the diet in our research did not affect fish growth parameters. The consequences of improved intestinal morphometric parameters are better absorption and utilization of nutrients; however, supplementation with fish diet prebiotics is still controversial, because improving intestinal morphometric parameters does not always have a positive effect on growth parameters and feed utilization, which was confirmed by Dimitroglou et al. [57,58], Salze et al. [59], and Zhou et al. [55]. This can be explained by the complex structure of the oligosaccharides used, the length of their feeding period, fish breeding conditions, or the method of preparation of feed [60,61,62].

Improving the morphological parameters of the intestine may contribute to the increase of fish immunity and consequently improved survival. Peterson et al. [61] showed that the addition of the prebiotic derived from a specific strain of *Saccharomyces cerevisiae* (Bio-Mos^®^) increased survival in channel catfish (*Ictalurus punctatus*), while weight gain and growth efficiency were similar. Improvement of intestinal morphology affects the maintenance of a healthy mucosal epithelium and defense against pathogenic bacteria [57]. This is due to the role played by goblet cells, which are specialized epithelial cells responsible for the secretion and distribution of mucins and that form a mucus layer, which has a protective function against mechanical and enzymatic damage to the intestine, as well as against pathogens. Torrecillas et al. [63], in their research on European sea bass (*Dicentrarchus labrax*), observed that prebiotics, including MOS (Bio-Mos^®^), may increase the secretion of mucus in the intestines. To improve the passage of food, the muscular layer responsible for intestinal peristalsis grows thicker, which was also observed in our own study.

## 5. Conclusions

The results of the present study revealed that prebiotics could be a potential dietary additive for farmed common carp. The supplementation of feed with 1% and 2% GOS significantly enhanced the development of the intestine, increased the height and width of the villi, and increased their surface area. In our study, the supplementation of a prebiotic had an effect on higher phosphorus absorption in the intestine. No differences in the biochemical blood parameters between the experimental groups may indicate maintenance of the metabolic balance of fish due to the prebiotic used. Similarly, the lack of changes in growth performance parameters under GOS treatment (in good rearing conditions) indicates that fish may be more resistant to potential deterioration of conditions or negative impacts of the environment and pathogens. The present study indicates that the prebiotic can be used as a feed supplement to modulate the intestinal histomorphology in common carp. Further research on the effects of prebiotics should be carried out, because fluctuations in hematological and biochemical variables may be associated with characteristics of species, inclusion rates of supplements, ingredients of diets, and rearing periods.

## Figures and Tables

**Table 1:** Dietary formulation and calculated nutritive values of experimental feeds.

Ingredient	Composition (%)		
	C	B1	B2
Fish meal ^1^	12.3	12.3	12.3
Blood meal ^2^	10.0	10.0	10.0
DDGS ^3^	11.0	11.0	11.0
Soybean meal ^4^	15.0	15.0	15.0
Rapeseed meal ^5^	10.0	10.0	10.0
Wheat meal	32.8	31.8	30.8
Fish oil ^6^	4.6	4.6	4.6
Soybean lecithin ^7^	1.0	1.0	1.0
Vitamin-mineral premix ^8^	1.5	1.5	1.5
Vitamin premix ^9^	0.1	0.1	0.1
Choline chloride	0.2	0.2	0.2
Fodder chalk	1.5	1.5	1.5
Prebiotic ^10^	0	1	2
Approximate composition (% dry matter)			
Crude protein	35.06		
Crude lipid	9.08		
Crude fibers	3.93		
Total phosphorus	0.83		
Calcium	1.36		
Ash	7.17		
Gross energy (MJ·kg^−1^)	18.51		
Essential amino acids	g/100 g of crude protein		
Arginine	4.53		
Histidine	2.8		
Lysine	3.5		
Tryptophan	1.04		
Phenylalanine + Tyrosine	4.96		
Methionine + Cysteine	1.75		
Threonine	3.13		
Leucine	6.72		
Isoleucine	3.9		
Valine	4.97		

^1^ Danish fishmeal, Type F, 72% total protein, 12% fat, FF Ska-gen, Denmark; ^2^ AP 301 P, 92% total protein, APC (GB) Ltd, Ings Road, Doncaster, UK; ^3^ Dried Distillers Grains with Solubles, >45% total protein, <6% ash; ^4^ Toasted, 46–47% total protein, 1% fat; ^5^ 33% total protein, 2% fat; ^6^ Agro-fish, Kartoszyno, Poland; ^7^ BergaPure, deoiled lecithin, 97% pure lecithin, Berg + SchmidtGmbH & Co. KG, Hamburg, Germany; ^8^ Polfamix W, BASF Polska Ltd. Kutno, Poland—1 kg contains: vitamin A 1,000,000 IU, vitamin D 3 200,000 IU, vitamin E 1.5 g, vitamin K 0.2 g, vitamin B 1 0.05 g, vitamin B 2 0.4 g, vitamin B 12 0.001 g, nicotinic acid 2.5 g, D-calcium pantothenate 1.0 g, choline chloride 7.5 g, folic acid 0.1 g, methionine 150.0 g, lysine 150.0 g, Fe 2.5 g, Mn 6.5 g, Cu 0.8 g, Co 0.04 g, Zn 4.0 g, J 0.008 g, carrier up to 1000.0 g; ^9^ Vitazol AD 3 E, BIOWET Drwalew, Poland—1 kg contains: vitamin A 50,000 IU, vitamin D 3 5000 IU, vitamin E 30.0 mg, vitamin C 100.0 mg; ^10^ Bitos^®^ trans-galactooligosaccharide (GOS), (Clasado Ltd, Reading, UK); dry powder containing a mixture (wt:wt) of the following oligosaccharides: 45% lactose, 9.9% disaccharides (Gal—(β1-3)—Glc; Gal—(β1-3)—Gal; Gal—(β1-6)—Gal; Gal—(α1-6)—Gal), 23.1% trisaccharides (Gal—(β1-6)—Gal—(β1-4)—Glc; Gal—(β1-3)—Gal—(β1-4)—Glc), 11.55% tetrasaccharides (Gal—(β1-6)—Gal—(β1-6)—Gal—(β1-4)—Glc), and 10.45% pentasaccharides (Gal—(β1-6)—Gal—(β1-6)—Gal—(β1-6)—Gal—(β1-4)—Glc).

**Table 2:** The effect of prebiotic Bi^2^tos^®^ on growth performance of common carp (*Cyprinus carpio* L.).

Items	Control *n* = 4	B1 *n* = 4	B2 *n* = 4	*p*-Value
FBW (g/fish)	503 ± 8.24	502 ± 23.2	503 ± 24.1	0.997
BWG(g/fish)	321 ± 8.86	319 ± 24.7	321 ± 24.7	0.988
FI(g/fish)	385 ± 3.01	388 ± 9.09	385 ± 9.60	0.636
FCR	1.22 ± 0.03	1.22 ± 0.07	1.20 ± 0.07	0.907
SGR(%/fish/day)	2.03 ± 0.04	2.02 ± 0.11	2.03 ± 0.11	0.962
PER	2.35 ± 0.06	2.35 ± 0.13	2.38 ± 0.14	0.897
PWG (%)	176 ± 6.12	174 ± 15.1	176 ± 14.4	0.967

FBW = final body weight; BWG = body weight gain; FI = feed intake; FCR = feed conversion ratio; SGR = specific growth rate; PER = protein efficiency ratio; PWG = percent weight gain.

**Table 3:** The effect of prebiotic Bi^2^tos^®^ on biochemical blood parameters in serum of common carp (*Cyprinus carpio* L.).

Items	Control *n* = 16	B1 *n* = 16	B2 *n* = 16	*p*-Value
TP (g/dL)	3.11 ± 0.32	3.09 ± 0.23	3.15 ± 0.35	0.838
Albumin (g/dL)	1.49 ± 0.11	1.50 ± 0.06	1.51 ± 0.06	0.891
Globulin (g/dL)	1.62 ± 0.26	1.59 ± 0.19	1.64 ± 0.31	0.871
Urea (mg/dL)	15.20 ± 1.98	17.53 ± 2.14	14.47 ± 4.50	0.538
TC (mg/dL)	148.88 ± 10.90	150.50 ± 12.46	153.38 ± 19.25	0.682
TG (mg/dL)	281 ± 99	348 ± 97	294 ± 88	0.122
NEFA (mmol/L)	0.31 ± 0.03	0.36 ± 0.03	0.31 ± 0.03	0.910
ALT (u/L)	25.75 ± 8.14	23.38 ± 6.91	20.44 ± 8.16	0.164
ALP (u/L)	116.19 ± 59.24	110.88 ± 66.21	78.88 ± 38.04	0.135
Ca (mg/dL)	11.06 ± 0.55	11.15 ± 0.71	10.88 ± 0.74	0.511
P (mg/dL)	12.37 ± 2.19 ^a,b^	13.62 ± 2.11 ^a^	11.81 ± 1.59 ^b^	0.039
Ca/P	0.92 ± 0.15	0.84 ± 0.16	0.93 ± 0.09	0.139
Glucose (mg/dL)	82 ± 15.71	75 ± 17.99	73 ± 13.69	0.118
Insulin (U/mL)	2.57 ± 0.33	2.60 ± 0.28	2.65 ± 0.13	0.854
TT_3_ hormone (nmol/L)	3.09 ± 1.29	4.58 ± 0.90	4.57 ± 1.90	0.138

Values marked with the different letters in the same line are significantly different (*p* ≤ 0.05 Tukey’s test). TP = total protein, TC = total cholesterol, TG = triglycerides, NEFA = non-esterified fatty acid, ALT = alanine aminotransferase, ALP = alkaline phosphatase, TT_3_ = thyroid hormone.

**Table 4:** The effect of prebiotic Bi2tos^®^ on histological measurements of intestines of common carp (*Cyprinus carpio* L.).

Items	Control *n* = 16	B1 *n* = 16	B2 *n* = 16	*p*-Value
Villi height VH (µm)	788.96 ^b^± 139.96	1040.98 ^a^ ± 159.65	981.21 ^a^ ± 152.58	<0.001
Villi width VW (µm)	121.71 ^b^ ± 14.98	144.35 ^a^ ± 18.65	150.05 ^a^ ± 14.17	<0.001
Villus surface VS (µm^2^)	300407.72 ^b^ ± 61527.71	471050,33 ^a^ ± 93853.46	462453,40 ^a^ ± 88447.20	<0.001
Crypt depth CD (µm)	175.39 ± 52.98	177.76 ± 25.97	148.15 ± 39.96	0.094
Tunica muscularis thickness (µm)	51.08 ^b^ ± 6.72	57.41 ^a,b^ ± 7.12	65.82 ^a^ ± 13.74	<0.001
Villi height/Crypt depth (VH/CD)	4.73 ^b^ ±1.15	6.03 ^a^ ± 1.56	6.91 ^a^ ± 1.46	<0.001

Values marked with the different letters (a,b) in the same line are significantly different (*p* ≤ 0.05. Tukey’s test).
